# Functional Cognitive Rehabilitation as a Primer to Activity-Based Stroke Telerehabilitation: Feasibility, Acceptability, and Engagement

**DOI:** 10.3390/brainsci15121298

**Published:** 2025-11-30

**Authors:** Stephanie Aghamoosa, Kelly Rishe, Julianne Laura, Patricia Finetto, Stephanie Garner, Lisa M. McTeague, Deena Schwen Blackett, Michelle L. Woodbury

**Affiliations:** 1 Department of Health Sciences and Research, College of Health Professions, Medical University of South Carolina, Charleston, SC 29425, USA; 2 Department of Psychiatry and Behavioral Sciences, College of Medicine, Medical University of South Carolina, Charleston, SC 29425, USA; 3 Ralph H. Johnson Veterans Affairs Medical Center, Charleston, SC 29401, USA; 4 School of Communication Sciences and Disorders, College of Health Professions and Sciences, University of Central Florida, Orlando, FL 32816, USA

**Keywords:** telerehabilitation, cognition, cognitive rehabilitation, occupational therapy, neuropsychology, stroke

## Abstract

**Background/Objectives**: Cognitive deficits are common after stroke and often compound motor impairments, hindering functional recovery—yet cognition remains under-addressed in stroke care. This pilot trial evaluated the feasibility and acceptability of a novel stroke telerehabilitation program—COG + OT—that delivers brief, functionally oriented cognitive rehabilitation as a primer to activity-based occupational therapy (OT). **Methods**: Twenty stroke survivors with arm/hand paresis participated in this single-arm pilot trial. The 8-week COG + OT program included 13 sessions across three phases: (1) cognitive rehabilitation (sessions 1–4), (2) application of cognitive strategies to task-practice OT (sessions 5–10), and (3) integration of cognitive skills into OT (sessions 11–13). Outcomes included feasibility (retention, adherence), acceptability (self-reported interest and usefulness of cognitive strategies, intervention acceptability), and engagement (digital literacy, barriers, and self-reported strategy use). **Results**: Retention was 95% and adherence was 99.6%. Participants reported moderate interest in cognitive strategies pre-intervention (M = 3.86/5) and found them moderately to very useful post-intervention (M = 4.42/5). Intervention acceptability and appropriateness were rated highly (M = 4.4/5). Common barriers included cognitive, environmental, and language factors; digital/technological barriers were infrequent. Self-reported use of cognitive strategies was moderate to high. **Conclusions**: The results of this trial strongly support the feasibility and acceptability of the COG + OT program for stroke survivors. Importantly, all participants were able to meaningfully engage in the program despite marked variability in cognitive and clinical characteristics. These findings support further investigation through randomized controlled trials to evaluate efficacy.

## 1. Introduction

Stroke is a leading cause of long-term disability [1,2], affecting both physical and cognitive function. Advances in acute stroke care have lowered mortality rates, resulting in more survivors living with chronic sequalae [3]. Among these, cognitive deficits are common and persistent—affecting 35–70% of stroke survivors and leading to dementia in up to 25% [4,5]. These impairments often last for years [6], even after clinical recovery [7], and compound physical symptoms (e.g., paresis, motor impairments), contributing to reduced daily function, participation, and quality of life [8,9]. Despite these impacts, cognitive symptoms remain under-addressed in stroke rehabilitation, which has traditionally focused on reductions in physical and/or language impairment. To address this gap, we developed a multidisciplinary, person-centered telerehabilitation program, which we call COG + OT, that integrates functional cognitive rehabilitation into an upper extremity task-practice telerehabilitation program [10].

Stroke often affects several cognitive domains, which may include attention, memory, executive functioning, language, and/or visuospatial abilities [11]. Together, these multi-domain cognitive deficits can result in poor functional cognition—the ability to use cognitive skills to perform everyday activities [12], thereby impeding functional recovery from stroke. Interventions that have been used to address post-stroke cognitive impairment vary widely in their approaches, ranging from targeted exercises for specific cognitive domains (e.g., computerized working memory training), to strategy training (e.g., mnemonic strategies to support memory), to individualized goal management training [13]. The term cognitive rehabilitation refers specifically to therapist-led interventions aimed at restoring or compensating for cognitive deficits in order to improve everyday functioning [14].

Cognitive rehabilitation is a generally effective tool for remediating cognitive dysfunction in stroke [15] that is within the scope of several disciplines including neuropsychology, speech–language pathology (SLP), and occupational therapy (OT). However, it remains largely absent from traditional stroke rehabilitation due to several barriers, including prioritization of physical impairments over cognitive and psychosocial issues [16], the siloing of cognitive interventions from other therapies, and limited access to cognition-focused specialized services (especially in underserved and rural communities [17]). Additionally, domain-specific approaches (e.g., attention training, compensatory memory strategies) have demonstrated limited generalization of gains to real-world function [15,18]. As such, evidence-based guidelines for cognitive rehabilitation in stroke increasingly emphasize metacognitive and “comprehensive-holistic” approaches to more optimally promote functional recovery [15]. This is the approach used in our new COG + OT telerehabilitation program, wherein the cognitive rehabilitation component is focused on functional cognition, which is essential for achieving activity-based goals during the upper extremity rehabilitation [19,20].

Intensive task-practice is the gold-standard stroke rehabilitation approach for addressing functional deficits during clinic-based rehabilitation [20,21,22]. The goal of an in-clinic task-practice session is for the stroke survivor to frequently practice an impaired task to elicit beneficial neural reorganization supporting functional recovery [23]. A newer OT rehabilitation model, the Cognitive Orientation to daily Occupational Performance (CO-OP) [24] approach, was developed to facilitate generalization of functional skill gains made during therapy to real-world situations. The CO-OP approach is based on learning-theory. With guidance and coaching from the therapist during task-practice, the individual learns metacognitive strategies to self-analyze performance, identify barriers, and problem-solve using cognitive strategies to improve performance [24,25]. Combining task-practice with the CO-OP approach has been shown to produce greater improvements in functional outcomes (both cognitive and motor) compared to task-practice alone [26]. However, because of the metacognitive demands inherent in the CO-OP approach, individuals with cognitive deficits may have difficulty with the CO-OP process.

Therefore, we developed the COG + OT program [10] to enable stroke survivors with cognitive deficits the opportunity to maximally engage with an OT program utilizing CO-OP task-practice methods. This innovative intervention delivers brief functional cognitive rehabilitation (COG) sessions prior to intensive CO-OP-focused task-practice led by an OT [27], such that the COG component serves as a “primer” for subsequent OT sessions. Priming is a term used to describe a stimulus that increases neuroplasticity in relevant networks, preparing the brain for engagement in a subsequent behavior (e.g., therapeutic task) [28]. The initial cognitive rehabilitation sessions focus on teaching compensatory strategies and leveraging cognitive strengths to address cognitive deficits. This cognitive rehabilitation component is designed to be personalized and functionally oriented (i.e., focused on the application of cognitive strategies to the individual’s daily activities), which is synergistic with the person-centered focus in the OT task-practice program. The cognitive strategies are then applied during subsequent OT sessions that gradually increase the complexity of the CO-OP metacognitive training. Thus, this priming design aims to level the playing field, enabling more stroke survivors—regardless of cognitive status—to fully engage in and benefit from this OT intervention.

We expect that our combined approach will enhance the ecological relevance of COG + OT and support more meaningful, real-world outcomes. Furthermore, delivering the intervention via telemedicine (i.e., telerehabilitation) is another innovative feature of our program. Both our group and others have successfully delivered functional task-practice and CO-OP training to stroke survivors via telerehabilitation [27,29,30]. More broadly, telerehabilitation has been shown to be an effective and feasible model across rehabilitation disciplines, producing outcomes comparable to those of in-person care with the added benefits of improved accessibility and reduced barriers [31,32,33,34]. These advantages further support the ecological validity of our approach, as telerehabilitation enables therapy delivery in real-world contexts where daily activities occur.

This manuscript reports the results of a pilot trial designed to assess the feasibility and acceptability of the COG + OT telerehabilitation program in a sample of 20 adult stroke survivors with arm/hand paresis. We examine recruitment and retention rates, adherence, and acceptability of both the novel cognitive components and the overall intervention. We hypothesized that COG + OT would demonstrate high feasibility—reflected by low attrition and strong adherence—and high acceptability among stroke survivors. In addition, we explore factors influencing engagement, including digital literacy, barriers encountered during sessions, and self-reported use of cognitive strategies. Together, these outcomes are essential first steps towards broader implementation and future trials evaluating the efficacy of COG + OT for improving functional, cognitive, and psychosocial outcomes after stroke.

## 2. Materials and Methods

### 2.1. Design

**Figure 1:**
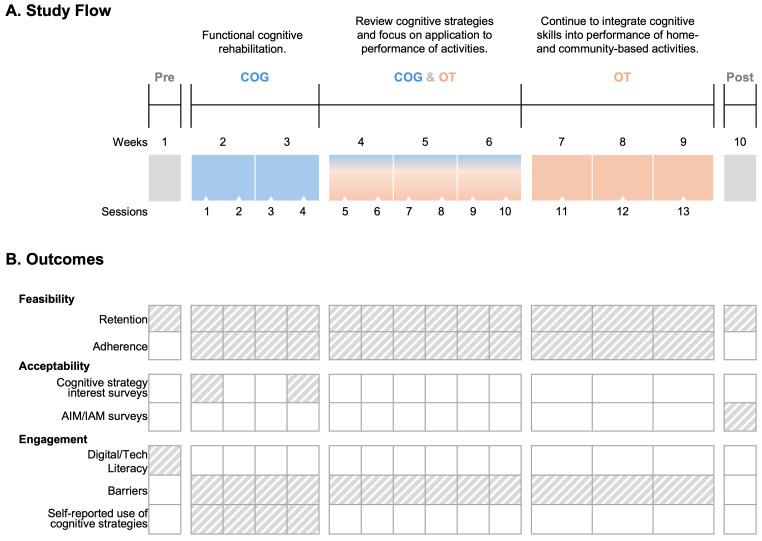
Study flow and outcome assessment timepoints. (**A**) Timeline of procedures conducted during each week of the study including outcomes assessments (“Pre” and “Post”) and descriptions of the three-phase intervention delivered across 13 sessions. (**B**) A list of outcome measures and depiction of collection timepoints (gray shading), corresponding to panel A.

This was a single group pilot trial of the COG + OT telerehabilitation program consisting of 13 intervention sessions over 8 weeks (Figure 1A) [10]. Pre- and post-treatment assessments were conducted within one week before and after the intervention. All assessment and treatment sessions occurred synchronously via a HIPAA-compliant videoconferencing platform (doxy.me) between a licensed OT (therapist; P.F., K.R., J.L., S.G.), who was experienced in stroke rehabilitation and specifically trained in telerehabilitation, and the participant in their chosen home or work environment. Study therapists were trained and supervised by the study Neuropsychologist (S.A.) and lead OT (M.L.W.). Research Electronic Data Capture (REDCap version 15.5.3) was used to securely collected and store all study data. This study was conducted at the Medical University of South Carolina between August 2024 and April 2025. This study was approved by the Institutional Review Board (Pro00122798) and registered with ClinicalTrials.gov (NCT06555302) prior to participant enrollment.

### 2.2. Implementation Considerations

We designed this study to pre-emptively address potential barriers. First, given the remote nature of the tele-sessions, we implemented procedures to ensure participant safety and minimize risks during the intervention. At the beginning of the study, the therapists gathered emergency response information including important contact information, designated emergency points of contact, the address at which telerehabilitation visits will occur, and information about any medical alert systems (e.g., wearable with fall detection). Each participant’s risk for falling during a telerehabilitation session was determined by the study therapist after collecting pertinent information at the pre-treatment assessment. This information included (1) confidence in balance/physical function, assessed via the self-reported Activities-Specific Balance Confidence (ABC) scale, which has established total score cut-points for stratification of function [35] (Table 1); (2) global cognitive function, measured with the Montreal Cognitive Assessment (MoCA [36]) (Table 1); and (3) relevant history ascertained via interview by the therapist (e.g., fall history, independence in daily routines, and availability of someone to assist with sessions). The therapist considered the combination of physical and cognitive functioning along with the history and contextual information to make a fall risk determination (i.e., minimal, moderate, or severe). If a participant was deemed a moderate or severe fall risk, additional precautions were taken to ensure safety. At-risk individuals were required to (a) have a second person (e.g., caregiver, family member, trusted friend) present for each telerehabilitation session to assist with balance/mobility during any dynamic standing activities and/or (b) complete the entire intervention in a seated position. Any adverse events were recorded by study therapists using standard forms, then reviewed by the clinician co-investigators.

Second, given that an estimated 30% of stroke survivors have aphasia, and these individuals are systematically excluded from most clinical research [37], we created “aphasia-friendly” materials [38] to enhance inclusivity. These materials were developed in consultation with and approved by the study team’s licensed SLP with stroke rehabilitation experience (D.S.B.). In line with best practices for this population [39], we used supportive communication strategies to facilitate comprehension and understanding, including (1) adaptive formatting (e.g., presenting small amounts of text at a time, increased spacing, larger font), (2) presenting information both verbally and visually, and (3) allowing participants to respond verbally, in writing, or by pointing/selecting from a multiple-choice list. We created aphasia-adapted visual aids for the informed consent document, the decision-making capacity assessment, and all pre- and post-treatment assessment measures. Additionally, the participant-facing worksheets used during COG sessions were written at an 8th grade reading level and incorporated visual aids. Although designed to support individuals with aphasia, the study therapists ultimately shared the adapted material with most participants as many found them helpful and supportive.

Third, we implemented a flexible study procedure timeline to increase adherence and retention by accommodating participants’ preferences and unforeseen conflicts. Visit schedules were set with consideration of participants’ preferences and other scheduled commitments (e.g., work, activities, appointments), fatigue management (physical and cognitive), and caregiver availability. Additionally, although all participants initially agreed to follow the pre-specified 8-week visit schedule, we allowed for make-up sessions to be added to any intervention week or extend into an additional 9th week when there were extenuating circumstances (e.g., serious weather events, illnesses, holidays). All intervention sessions were completed in sequence regardless of visit schedule modifications.

### 2.3. Participants

We recruited N = 20 individuals from a stroke research registry to participate in this study. This sample size was selected based on recommendations for feasibility studies, which emphasize pragmatic sample sizes (typically 10–40 participants) to assess recruitment, retention, and adherence rather than statistical power for efficacy testing [40]. This approach aligns with the primary objectives of our pilot trial, which are to establish feasibility and acceptability of the COG + OT intervention before progressing to larger efficacy studies.

**Figure 2:**
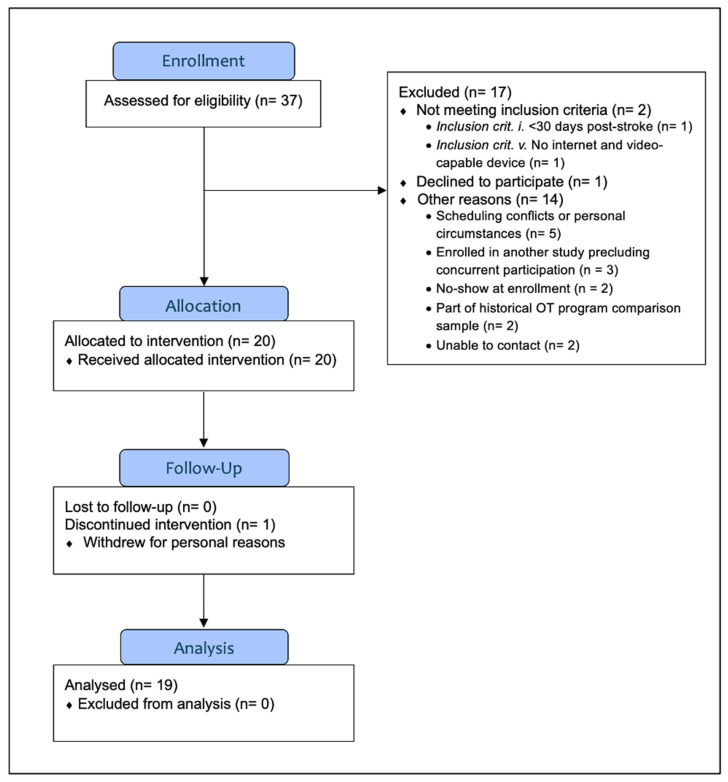
CONSORT diagram.

The inclusion criteria were as follows: individuals who (i) had experienced ischemic or hemorrhagic stroke with resultant paresis of one arm/hand at least 30 days prior; (ii) were adults age 21 years or older; (iii) were able to speak and read English; (iv) had corrected vision to be able to read text on a screen; (v) had a device on which a telerehabilitation visit can be conducted (e.g., phone, tablet, laptop) and a Wi-Fi connection or cellular service; (vi) were able to participate in the study’s assessment sessions as per the therapist’s judgment. Participants were excluded if they had (i) moderate-severe or severe aphasia or (ii) impaired decision-making capacity. To screen for aphasia, participants were first asked if they had difficulty communicating with others since their stroke; if self-reported problems were endorsed, they were then administered the Language Screening Test (LAST) [41] and excluded if they scored below <6/7 on the receptive index or <4/8 on the expressive index. Participants were also excluded if they scored below 15/15 on the LAST (but above the receptive/expressive cutoffs) and were deemed ineligible by the OT and study SLP (D.S.B.) due to severity of aphasia. Three potential participants endorsed communication difficulties; however, all scored 15/15 on the LAST and were therefore deemed eligible. We used the Understanding, Appreciation, Reasoning, and Expression (U-ARE) protocol [42] for assessing capacity to provide informed consent. No participants were excluded based on the U-ARE protocol. The CONSORT diagram (Figure 2) presents the number of individuals who were assessed for eligibility, excluded based on the eligibility criteria listed above, enrolled, and completed the study. Descriptive statistics for participant demographics and characteristics are presented in Table 1.

### 2.4. Intervention

Intervention development and procedures are described previously [10]. We used a titrated design to first deliver brief functionally oriented COG as a primer to an existing telerehabilitation OT program. The intervention had three phases: (1) COG in sessions 1–4, (2) hybrid COG + OT in sessions 5–10, and (3) OT sessions with continued application of cognitive strategies in sessions 11–13 (Figure 1A). To ensure consistent delivery of the intervention content across study therapists, all sessions followed a standardized structure. Each session began with a brief review of content covered in the previous session and the assigned homework, followed by the introduction of new content, and ending with a brief review and assignment of new homework. This structure was built into the daily treatment note for each session in REDCap which specified allotted time per segment, conversation prompts, and documentation fields. All therapists used the treatment notes to guide session flow, collect in-session data, and document progress, and these data were routinely reviewed to ensure fidelity.

The COG session materials were adapted from an existing 8-week manualized cognitive rehabilitation program [43] and covered the following content: Session (1) Introduction, Cognitive and Lifestyle Factors, Basic Attention (strategies: taking breaks, self-talk), Session (2) Attention and Concentration (strategies: active listening, environmental adjustments), Session (3) Organization and Memory (strategies: lists, reminders, linking tasks, automatic places, visual imagery), Session (4) Problem Solving and Routines (strategies: systems, prioritizing, planning). These domains were chosen because they are frequently impaired after stroke [11] and have direct implications for the ability to manage everyday activities and roles (i.e., functional cognition). For example, deficits in attention, memory, problem-solving, and organization can interfere with completing complex or multi-step tasks such as medication management, meal preparation, and financial management [44,45]. Because cognitive impairment was not a requirement for enrolling in this study, COG session content was intentionally flexible in its focus on improving function (for participants experiencing cognitive difficulties) vs. optimizing skills (for those who felt they were functioning well). For each COG session, we provided study-developed 1–2-page worksheets via screen-sharing and/or email to guide discussion and as a centralized space for taking notes, which participants and caregivers were encouraged to review between sessions. Additionally, therapists could reference supplemental session guides developed for each COG session that included additional examples, activities, and prompts.

The telerehabilitation OT program was developed in prior research [27] and consists of 9 sessions of upper-extremity task-practice targeting each individual’s ability level [46,47] and metacognitive training using CO-OP [24]. In the current study, the initial OT sessions (5–10) were hybrid COG + OT, which involved reviewing previously learned cognitive strategies at the beginning of each session and explicit application of those strategies to the OT activities. The final OT sessions (11–13) focused on continued integration of cognitive strategies into OT activities.

All COG and OT material was delivered in an individualized manner, focusing most on the content of highest relevance to each participant’s goals and functional level. Homework was collaboratively decided upon between the therapist and participant at the end of each session.

### 2.5. Measures

All measures were administered via telehealth (HIPAA-compliant videoconferencing platform, doxy.me) by the study OTs. All data were collected and stored within REDCap, a secure, web-based application designed to support data capture for research studies. The full list of outcome measures (i.e., feasibility/acceptability, primary, and secondary) for this trial are described previously [10]. In the current paper, we are reporting results of the feasibility and acceptability outcome measures, which we describe below. The timepoints for assessment of each of these outcomes are depicted in Figure 1B.

Sample Characterization: The following measures were collected at baseline by the study therapists who were trained raters. Global cognition was measured using the MoCA [36], a 16-item screening measure with scores ranging from 0 to 30 points where higher scores indicate better cognition. Motor impairment was measured using the Fugl-Meyer Assessment of the Upper Extremity [48] modified for use in telerehabilitation (tFMA-UE), as in our prior work [27]. The tFMA-UE has 22 items pertaining to observed upper extremity motor ability, each rated by the therapist on a 3-point scale (0 = unable to perform, 1 = partially performed, 2 = near normal performance). The tFMA-UE scores range from 0 to 44, with lower scores indicating more motor impairment.

Feasibility:. Retention was measured as the percentage of enrolled participants who completed all study procedures. Adherence was measured as the percentage of intervention sessions (out of 13) completed.

Acceptability: Cognitive Strategy Interest Surveys were administered to measure participants’ interest in learning cognitive strategies prior to beginning COG (beginning of session 1) and the perceived usefulness of the strategies after COG (end of session 4). For each of 4 cognitive domains—Attention, Memory, Organization, Problem Solving—participants were asked, “How interested are you in learning strategies for each cognitive skill listed below?” (session 1) and “How useful were the cognitive strategies you learned for improving each area of thinking listed below?” (session 4). Ratings were on a 5-point Likert scale (1: not interested/ useful at all, 3: somewhat interested/useful, 5: very interested/useful) or 0 (I don’t know what this is). Participants also had the option to provide narrative comments.

**Figure 3:**
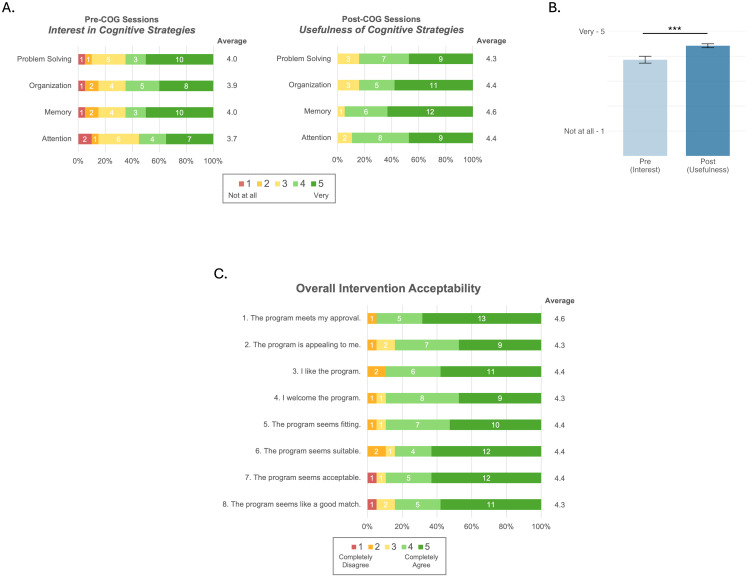
Participant ratings of intervention acceptability. (**A**) Stacked bar charts show ratings of participants’ interest in learning cognitive strategies in each of the four domains (y-axis) prior to beginning COG sessions (**left**) and the perceived usefulness of the strategies in each domain after completing COG sessions (**right**). Ratings range from 1 = Not at all (red) to 5 = Very (dark green). (**B**) Using the same data, the bar graph shows the mean ratings ± SE (y-axis) across all four strategies at each timepoint (x-axis). Interest in learning the cognitive strategies pre-intervention was significantly lower than perceived usefulness of the cognitive strategies after completing the COG component of the intervention. (**C**) Stacked bar chart shows ratings on the Acceptability of Intervention Measure (AIM; items 1–4) and Intervention Appropriateness Measure (IAM; items 5–8). Ratings range from 1 = Completely Disagree (red) to 5 = Completely Agree (dark green). Note. *** *p* < 0.001.

Intervention Experience Surveys were administered at the post-treatment visit to assess participant-rated intervention acceptability (Acceptability of Intervention Measure; AIM) and appropriateness (Intervention Appropriateness Measure; IAM) [49]. To avoid response bias arising from having a therapist present, participants were sent a link to complete the surveys independently and/or with the assistance of a caregiver. However, if the participant had difficulty completing the surveys and a caregiver was not available, they could be assisted by the study therapist. The AIM and IAM each had four statements (listed in Figure 3C), which participants rated on a 5-point Likert scale (1: completely disagree, 2: disagree, 3: neither agree nor disagree, 4: agree, 5: completely agree). Participants were also asked to provide narrative responses to what they liked and did not like about the program.

Engagement: Digital/technology literacy was rated by the study therapist at the conclusion of the first pre-treatment visit using a set of questions developed by our team that query the participant’s ability to understand and use various technological functions required for engaging in the tele-sessions and the amounts/types of support needed. These ratings were informed by the therapist’s observations and interactions with the participant and/or caregiver during that first visit.

Barriers encountered during each intervention session were recorded by the therapist selecting one or multiple options from the following list: “None”, “Caregiver-Related Barriers (time, engagement, etc.)”, “Cognition”, “Safety”, “Participant compliance with home program”, “Digital/Tech Literacy (participant’s ability to interact with therapist and telerehabilitation platform)”, “Device Issues (technology failures, connectivity issues, etc.)”, “Environmental Limitations (home set-up, access to functional spaces, etc.)”, “Language/Communication”, “Other” (therapist provided description). For sessions involving OT (sessions 5–13), there were two additional options: “Low motor function”, “High motor function”. To summarize barriers, we first separated the responses by session-type: COG-only sessions (sessions 1–4) vs. all others (sessions 5–13). Then, within each session-type, we computed a percentage by dividing the number of times each barrier was recorded by the total number of sessions (i.e., sum of all sessions across all participants).

Self-reported use of cognitive strategies was measured during the COG phase of the intervention (sessions 1–4). At the end of each COG session, participants were asked, “How much do you think you will use the strategies we discussed today?” At the beginning of the subsequent COG session, they were asked, “Since our last session, how much did you use the cognitive strategies we learned in the last session?”. Ratings were on a 5-point Likert scale (1: never, will/did not use the strategies, 3: sometimes, 5: very often).

### 2.6. Statistical Analyses

Statistical analyses were conducted in R version 4.4.1. We report descriptive statistics for sample demographics, clinical characteristics, and all feasibility, acceptability, and engagement outcomes. For the acceptability outcomes from the Cognitive Strategy Interest Surveys, we conducted a two-way analysis of variance (ANOVA) to examine whether participants’ ratings varied by cognitive strategy type (i.e., Problem Solving, Organization, Memory, and Attention), timepoint (pre-COG sessions [interest] vs. post-COG sessions [perceived usefulness]), and their interaction. For the engagement outcome self-reported cognitive strategy use, we conducted a two-way ANOVA to examine the effects of rating type (anticipated vs. actual use), COG session (Session 1: Basic Attention, Session 2: Attention and Concentration, Session 3: Organization and Memory, Session 4: Problem Solving and Routines), and their interaction on participants’ ratings of strategy use. For ANOVA models, we report effect sizes using partial eta squared (η^2^_p_) along with 95% confidence intervals (CI). The one participant who discontinued the intervention was excluded from analysis due to incomplete data. One other participant was missing a single rating (Session 2 anticipated strategy use); all other data were complete.

## 3. Results

We enrolled 20 adult stroke survivors into this trial. This sample ranged in age from 36 to 75 (M = 57.6, SD = 12) and was 50% female, 65% White, and 95% Non-Hispanic/Latino (see Table 1 for full demographic and clinical characteristics). Given that cognitive impairment was not a prerequisite for enrollment in the study, scores on the MoCA global cognitive screener ranged from impaired to intact (range = 16–29) and were mildly impaired on average (M = 23.8). Upper extremity motor impairment in this sample ranged from severe to mild (tFMA-UE score range = 0–41), with moderate/mild impairment on average (M = 27.9).

### 3.1. Safety

Over half of the participants (*n* = 12, 60%) were determined by the therapists to have minimal fall risk, requiring no modifications to the OT sessions. Of the remaining participants (*n* = 8), all but one had caregivers present during OT sessions to ensure safety (caregiver types listed in Table 1), and the individual with no caregiver completed seated activities only. There were no serious adverse events during the study. There were six adverse events reported, three of which were not related to the intervention. Two were deemed possibly related to the intervention: (1) bruise on foot from engaging in exercise not prescribed by the study therapist (mild severity) and (2) elbow bursitis on affected upper extremity (moderate severity). One was deemed “definitely related” to the intervention: pain in the affected upper extremity (elbow) during OT session (mild severity). All participants who experienced adverse events were able to complete the remainder of the intervention sessions.

### 3.2. Feasibility

Retention was 95.0%, with 19 of 20 treatment initiators completing all study procedures (Figure 2). The one participant who withdrew from the study had completed the first week of the intervention (two COG sessions) and chose to discontinue due to conflicting personal obligations. Among the 19 treatment completers, adherence was 99.6% (only one final OT session was not completed). This participant needed to truncate intervention sessions due to beginning another medical treatment for a non-stroke-related comorbidity but still wished to complete this study. The therapist modified the intervention schedule to cover all material in 12 sessions rather than 13.

### 3.3. Acceptability

Participants were asked to rate their interest in learning cognitive strategies prior to beginning the first COG session and then rate the perceived usefulness of the strategies after the final COG session (Figure 3A). A two-way ANOVA revealed a significant main effect of time such that average interest ratings collected prior to starting COG sessions (M = 3.86, SD = 1.24) were lower than the average perceived usefulness ratings collected after completing all 4 COG sessions (M = 4.42, SD = 0.70; F(1, 148) = 11.55, *p* < 0.001, η^2^_p_ = 0.07, 95% CI [0.02, 1.0]; Figure 3B). Ratings of interest and perceived usefulness did not differ across strategy types overall (F(3, 148) = 0.41, *p* = 0.745, η^2^_p_ = 0.008, 95% CI [0.0, 1.0]), and there was no significant time × strategy interaction (F(3, 148) = 0.28, *p* = 0.842, η^2^_p_ = 0.006, 95% CI [0.0, 1.0]). Descriptively, participants reported being somewhat-to-moderately interested in all cognitive strategy domains (range: 3.7–4/5), the highest being Memory and Problem Solving, followed by Organization, and lastly Attention. After completing the COG sessions, participants found all strategies moderately useful (range: 4.3–4.6/5), the most useful being Memory, followed by Organization and Attention, and lastly Problem Solving. All participants who completed the intervention (*n* = 19) completed the AIM and IAM measures of intervention acceptability and appropriateness. Of those, 15 (79%) were completed without the study therapist’s assistance. Participants provided favorable ratings of overall intervention acceptability (AIM: M = 4.4/5) and appropriateness (IAM: M = 4.4/5; Figure 3C).

### 3.4. Engagement

**Figure 4:**
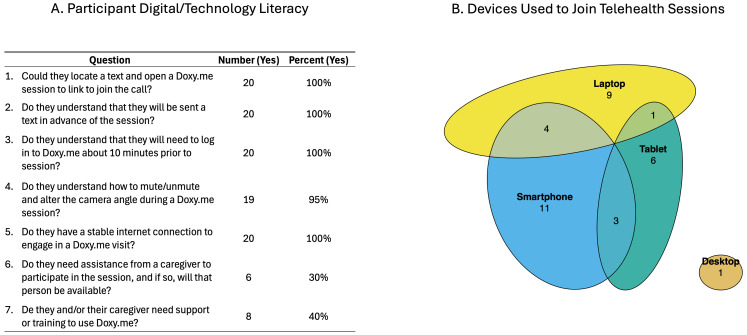
Digital and technology factors. (**A**) The table summarizes the study therapists’ ratings of participants’ digital/technology literacy. (**B**) The Venn diagram depicts the number of participants using each device type to engage in the intervention sessions; numbers within overlapping segments indicate the use of more than one device.

Digital/technology literacy at enrollment was rated positively (Figure 4), with all participants having sufficient internet connectivity and a good understanding of and ability to complete the steps for joining the video visits. Of the 20 treatment initiators, 6 (30%) needed assistance from a caregiver to participate in virtual visits and 8 (40%) needed some level of support or training to initiate use of the videoconferencing platform at the beginning of the study.

**Figure 5:**
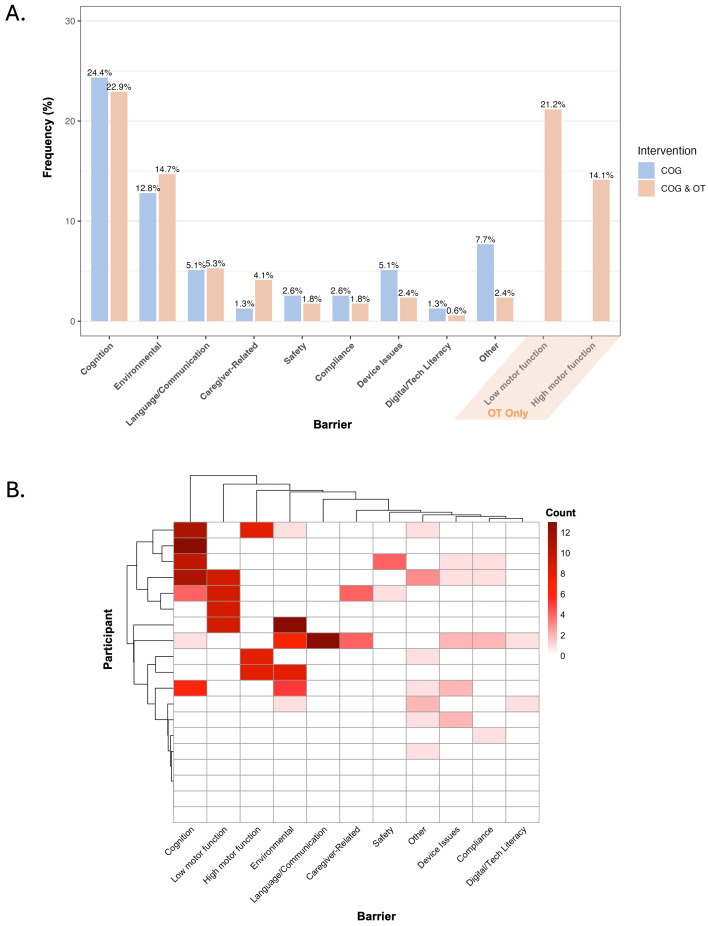
Barriers to engagement in the intervention. (**A**) Bar graph shows the percentage of total sessions (i.e., frequency; y-axis) in which each barrier type (bar pairs; x-axis) was reported, aggregated across participants. For each barrier type, the percentage is reported for the COG-only intervention sessions (sessions 1–4; blue bar, left of pair) and the remaining COG + OT sessions (sessions 5–13; orange bar, right of pair). The final two barrier types (low/high motor function) were not queried during COG-only sessions, so only orange bars are presented. (**B**) Heatmap depicts the total number of times each barrier (columns) was reported by each participant (rows) across all intervention sessions. The heatmap was generated using the “pheatmap” function in R. Hierarchical clustering was applied to both rows (participants) and columns (barriers) to visualize patterns of similarity, using default clustering parameters.

The frequencies of therapist-reported barriers encountered during the intervention, reported separately for COG only and COG + OT sessions, are presented in Figure 5. Cognition and environmental limitations (e.g., home set-up, access to functional spaces) were common barriers for both session types. Whereas environmental limitations were reported more frequently in OT sessions than COG sessions, cognition posed the largest challenge during COG sessions. Caregiver-related barriers (e.g., time, engagement) came up more often in OT sessions, reflecting the greater need for caregiver involvement in this portion of the intervention to ensure participant safety. Despite some participants needing initial training in technology use at the pre-treatment visit, digital/technological barriers were relatively infrequent during the intervention sessions (Figure 5).

**Figure 6:**
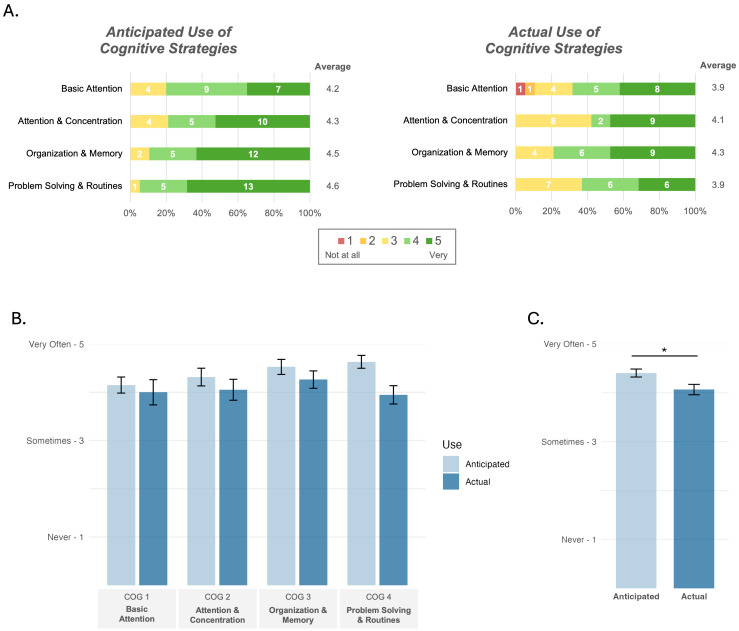
Self-reported cognitive strategy use. (**A**) Stacked bar charts show the distribution of participant’s ratings of anticipated (**left**) and actual (**right**) use of the cognitive strategies presented in each session (y-axis), ranging from 1 = Never (red) to 5 = Very Often (dark green). Average rations across the sample are presented to the right of each chart. Using the same data, the bar graphs show the mean ratings ± SE (y-axes) for (**B**) anticipated use (light blue, left of pair) and actual use (dark blue, right of pair) across sessions (x-axis) and (**C**) the significant difference between average ratings across sessions for anticipated vs. actual use of cognitive strategies (x-axis). Note. * *p* < 0.05.

Self-reported ratings of anticipated and actual use of cognitive strategies learned during each COG session are presented in Figure 6. Average ratings for both anticipated and actual cognitive strategy use were favorable (range: 3.95–4.63/5; 5 = “very often”). The results of the two-way ANOVA indicated a significant main effect of rating type such that average ratings for anticipated use (M = 4.40, SD = 0.73) were higher than actual use (M = 4.06, SD = 0.95; F(1, 146) = 6.08, *p* = 0.015, η^2^_p_ = 0.04, 95% CI [0.0, 1.0]; Figure 6C). Use ratings did not differ across sessions (F(3, 146) = 1.02, *p* = 0.387, η^2^_p_ = 0.02, 95% CI [0.0, 1.0]), and there was no significant rating type × session interaction (F(3, 146) = 0.74, *p* = 0.531, η^2^_p_ = 0.01, 95% CI [0.0, 1.0]; Figure 6B), suggesting that participants did not rate the anticipated or actual use of any cognitive strategy more highly than others.

## 4. Discussion

We conducted a pilot trial of the COG + OT program, a new telehealth-delivered OT-led intervention that integrates functional cognitive rehabilitation as a primer to an upper extremity-focused, task-practice stroke rehabilitation program. COG + OT was designed to be a whole-person telerehabilitation program that is not only responsive to both cognitive and physical needs, but also personalized, flexible, scalable, and readily translatable to clinical practice. Our feasibility and acceptability data support these aspects of the program. We achieved high rates of retention and adherence to this 8-week intervention, and participants rated both the cognitive rehabilitation components (first 4 sessions) and the overall program favorably. Importantly, despite there being considerable heterogeneity in participants’ clinical characteristics, cognitive status, and physical functioning, all were able to meaningfully engage in the telerehabilitation program.

There was clear interest in and demand for this telerehabilitation program, as evidenced by our ability to easily enroll 20 stroke survivors within just 8 months. Moreover, only one enrollee discontinued participation, and only one intervention session was otherwise not completed (99.6% adherence), which we largely attribute to the flexibility afforded by telehealth. Notably, adherence to COG + OT was slightly higher than adherence rates reported in recent at-home stroke rehabilitation intervention studies [50,51]. To ensure broad applicability, we enrolled a heterogeneous sample of stroke survivors with varying cognitive status (from impaired to intact) and upper extremity motor function (from low to high). Despite this variability, all participants were able to identify functional goals in collaboration with the study therapist, and the program was rated as highly acceptable and appropriate.

This success is particularly notable for the 4-session COG component given that cognitive impairment was not required for enrollment. A major challenge in addressing post-stroke cognitive deficits is the variability in affected domains, severity, and interaction with physical impairments, all having potentially different effects on the individual’s daily function [11]. Despite this variability, participants rated the cognitive strategies favorably across multiple domains—interest, perceived usefulness, anticipated use, and actual use. Furthermore, participants reported strong interest in learning all cognitive strategies prior to the training, and subsequently rated the perceived usefulness of the strategies after learning them even higher. This suggests that participants may have gained a deeper appreciation for the strategies’ functional relevance through engagement with the intervention. We also found that participants’ prospective ratings of anticipated strategy use were significantly higher than their retrospective ratings of actual strategy use, although the difference was numerically small (anticipated: 4.4/5 vs. 4/5, where 5 = “very often”). We interpret this difference as likely reflecting participants’ high motivation and intent to use the strategies as well as honest reporting of their moderate success. Importantly, there were no differences across the cognitive domains (i.e., attention, memory, organization, problem solving) for all ratings, highlighting that the content covered in the cognitive rehabilitation sessions was broadly applicable regardless of individual differences. This likely reflects the personalized and functionally oriented cognitive rehabilitation approach used in this study.

In designing the COG component, we aimed to address functional cognition by integrating strategies across cognitive domains and demonstrating their relevance to daily life [12]. Our intention was to deliver the content in a way that was highly personalized, this being a key strategy for increasing motivation in stroke rehabilitation [52]. To achieve this, the patient-facing worksheets included reflection prompts and therapists were trained to work collaboratively with participants to identify how relevant strategies relate directly to their daily activities. Further, the material was designed to support both remediation of impaired cognitive abilitites and optimization of intact ones. The success of this approach is supported by the self-reported use ratings, which indicated strong intentions to use the strategies (anticipated use) and successful transfer and application of the strategies to their everyday lives (actual use). Together, these findings highlight that strategies focused on improving functional cognition are appealing, engaging, useful, and ecologically relevant for stroke survivors.

Although cognitive rehabilitation has traditionally been siloed [15], there is growing recognition of its importance to stroke rehabilitation [53] and the need to address functional cognition as a core component of recovery [54]. In line with this view, we believe that an ideal cognitive rehabilitation program for stroke survivors should be integrated into, rather than separated from, a physical rehabilitation program to efficiently address multi-domain care needs and synergistically enhance outcomes. This rationale informed our use of priming in COG + OT, wherein cognitive strategies were introduced first and then reinforced during subsequent OT sessions. Integrating functionally oriented cognitive rehabilitation in this way is a natural extension of the OT-led CO-OP task-practice approach used in this program [55]. Importantly, stroke survivors in this study found the overall program highly acceptable despite the addition of four COG sessions. We anticipate that this approach may amplify the benefits previously observed from task-practice alone [27], which will be evaluated in a forthcoming paper.

The results of this trial underscore a key strength of the intervention—its potential for clinical translation. The 8-week timeline is feasible within current outpatient reimbursement models [56]. Additionally, as emphasized by the World Health Organization [57], telehealth delivery may improve accessibility for individuals facing transportation or logistical barriers. With easily implemented fall risk assessments and precautionary procedures (e.g., caregiver presence, seated activities), we maintained participant safety and comfort during virtual sessions. Digital literacy and telehealth delivery were not significant barriers; any technology-related issues were easily addressed through brief training and assistance by the study therapists. The most frequent barriers were related to cognitive, environmental, and language/communication factors (all occurring in less than a third of sessions), and motor function for the OT portion specifically. Importantly, none of these barriers prevented successful participation. These findings showcase our successful strategies addressing critical telerehabilitation competencies [58].

This pilot trial has several limitations and implications for future research. Our sample size (N = 20) was chosen to assess feasibility and acceptability [40], but its small size limits representativeness and generalizability. Participants’ motor impairment ranged from mild to severe, similar to large stroke rehabilitation trials with similar motor inclusion criteria such as the ICARE trial [59]. However, we did not assess factors influencing motor skill, such as sensation, spasticity, strength or vision, thus limiting the generalizability of our results. Because cognitive complaints or impairments were not required for enrollment, findings may not generalize to samples with diagnosed cognitive disorders or severe impairments, which are common post-stroke [4,5]. Although some individuals had low MoCA scores (minimum = 16/30), replication in more cognitively impaired samples is warranted. Relatedly, future studies should include more comprehensive cognitive measures beyond a global screener (i.e., MoCA). Specifically, a neuropsychological test battery would provide improved sensitivity to subtle deficits and more detailed characterization of cognitive profiles, and a measure of functional cognition, which could include self-report or performance-based measures [12], would directly measure this construct. Additionally, this study did not measure anosognosia—limited awareness of one’s impairments—which is a common post-stroke symptom [60,61] that could influence engagement and perceived benefit. Including such measures will help determine how feasibility and acceptability vary across cognitive characteristics.

## 5. Conclusions

This pilot trial supports the feasibility of delivering COG + OT to stroke survivors—a telerehabilitation program that uses brief, functionally oriented cognitive rehabilitation as a primer to activity-based OT. These findings justify future randomized controlled trials to evaluate the efficacy of COG + OT. If found effective, subsequent research may explore differential effects across cognitive severities and profiles and identify predictors of treatment response to guide intervention refinement. This line of work is critically needed, as successfully addressing post-stroke cognitive symptoms may substantially reduce the risk of subsequent decline and dementia [6], enhance rehabilitation outcomes, and improve daily function.

## Figures and Tables

**Table 1:** Sample demographics and characteristics (*N* = 20 treatment initiators).

Category	Measure	Mean or Number	SD or %	Range
Demographics	Age (years)	57.6	12	36–75
	Education (>12 years)	17	85%	
	Sex (Female)	10	50%	
	Gender (Female)	10	50%	
	Race			
	White	13	65%	
	Black or African American	5	25%	
	Native Hawaiian or Other Pacific Islander	1	5%	
	Other	1	5%	
	Ethnicity (Not Hispanic/Latino)	19	95%	
Clinical Characteristics	Time Since Stroke (years)	3.8	3.4	0.4–14
	Hemisphere			
	Right	14	70%	
	Left	5	25%	
	Bilateral	1	5%	
	Affected side			
	Right	9	45%	
	Left	9	45%	
	Bilateral	2	10%	
	Stroke Subtype			
	Acute Ischemic	16	80%	
	Intracranial Hemorrhage	3	15%	
	Subarachnoid Hemorrhage	1	5%	
	Receiving Other OT Services			
	No	17	85%	
	Yes (Outpatient)	3	15%	
Baseline Measures	Upper Extremity Function (tFMA-UE total score)	27.9	12.7	0–41
	Depression (PHQ-9 total score)	5.2	5.0	0–16
	Cognition (MoCA total score)	23.8	3.6	16–29
	Balance Confidence (ABC Scale total score)	65.2	24.2	21–99
	Functional Stratification (ABC Scale cut-points)			
	High Functioning (≥80)	6	30%	
	Moderate Functioning (50–79)	9	45%	
	Low Functioning (<50)	5	25%	
	Fall Risk (Clinician-Determined)			
	Minimal	12	60%	
	Moderate	2	10%	
	High	6	30%	
	Caregiver present			
	Spouse/partner	6	60%	
	Adult Child	1	10%	
	Family (other)	1	10%	
	Friend	1	10%	
	Hired Caregiver	1	10%	

## Data Availability

The data presented in this study are available on request from the corresponding author due to privacy reasons.

## Abbreviations

The following abbreviations are used in this manuscript:

COG	Cognitive rehabilitation
OT	Occupational therapy/ist
SLP	Speech–language pathology/ist
TOT	Task-oriented training
CO-OP	Cognitive Orientation to daily Occupational Performance
ABC	Activities-Specific Balance Confidence
MoCA	Montreal Cognitive Assessment
LAST	Language Screening Test
U-ARE	Understanding, Appreciation, Reasoning, and Expression protocol
CONSORT	Consolidated Standards of Reporting Trails
AIM	Acceptability of Intervention Measure
IAM	Intervention Appropriateness Measure
tFMA-UE	Telerehabilitation version of the Fugl-Meyer Assessment of the Upper Extremity
ANOVA	Analysis of variance
